# Organic Turkey Flocks: A Reservoir of *Streptococcus gallolyticus subspecies gallolyticus*

**DOI:** 10.1371/journal.pone.0144412

**Published:** 2015-12-10

**Authors:** Jochen Schulz, Jessika Dumke, Dennis Hinse, Jens Dreier, Christin Habig, Nicole Kemper

**Affiliations:** 1 Institute for Animal Hygiene, Animal Welfare and Farm Animal Behaviour, University of Veterinary Medicine Hannover, Foundation, Hannover, Germany; 2 Institute for Laboratory and Transfusion Medicine, Ruhr University of Bochum, Bad Oeynhausen, Germany; Centers for Disease Control & Prevention, UNITED STATES

## Abstract

*Streptococcus gallolyticus* subspecies *gallolyticus* (*S*. *gallolyticus*) can colonise the gastrointestinal tract of humans and animals and is known to cause similar infections in both humans and animals. Data about the spread or prevalence in farm animals are missing. In this study, Trypton Soya Agar was modified to a selective medium enabling the isolation and quantification of *S*. *gallolyticus* from faecal samples. The bacterium was observed in 82 out of 91 faecal samples obtained from 18 different organic turkey flocks. The prevalence of shedding birds was estimated by the number of positive fresh droppings and reached up to 100% on most farms. Furthermore, for the first time *S*. *gallolyticus* was quantified in faeces from poultry flocks. The median of colony forming units (CFU) per gramme faeces was 3.6 x 10^5^CFU/g. Typing of one isolate from each positive faecal sample by multilocus sequence typing delivered 24 sequence types (STs). Most of the isolates belonged to the clonal complex CC58. The same STs of this complex were detected in up to six different flocks. Partly, these flocks were located in various regions and stocked with varying breeding lines. Regarding the biochemical profiles of the same STs from different farms, the results did not contradict a spread of specific STs in the organic turkey production. Moreover, checking the pubMLST database revealed that STs found in this study were also found in other animal species and in humans. The high detection rate and the number of *S*. *gallolyticus* in turkey faeces indicate that this bacterium probably belongs to the common microbiota of the gastrointestinal tract of turkeys from organic flocks. Furthermore, the findings of this study support the suggestion of a possible interspecies transmission.

## Introduction

*Streptococcus gallolyticus* subspecies *gallolyticus*, formerly named *Streptococcus bovis* biotype I and hereinafter referred to as *S*. *gallolyticus*, can be a part of the gut microbiota of wild animals, companion animals, farm animals and humans [1–5]. This species can be an opportunistic pathogen in humans and animals. In humans, for instance, *S*. *gallolyticus* can cause bacteremia, sepsis, meningitis, and endocarditis [5]. Interestingly, the latter named infection seems to be associated with colorectal cancer [6, 7]. For animals, infections such as septicaemia, subclinical intramammary infection, gross lesions of the liver and spleen and endocarditis are described [8–11]. Although *S*. *gallolyticus* has been considered as relatively unimportant in veterinary medicine so far, it can be an important agent of streptococcosis in pigeons [12] and case reports and outbreaks with increased mortalities in turkeys, laying hens and broiler flocks have been published [2, 10, 13]. Moreover, due to similar infections in humans and animals there is an ongoing discussion regarding the zoonotic potential of *S*. *gallolyticus*. For example, 50% of human clinical isolates previously designated as *Streptococcus bovis* were reclassified to *S*. *gallolyticus*[14]. Furthermore, *S*. *gallolyticus* became the first cause of infectious endocarditis among streptococci in humans from Europe [15]. To date, it is not clear if infections in humans are solely caused by strains which colonise humans only or if a transmission from an animal to a human is possible. This lack of knowledge is probably related to the genetic heterogeneity of infectious strains, the kind of applied genotypic characterisations [16, 17] and to the limited number of characterised isolates from people with close animal contact. Concerning the latter point it was shown for instance that the close contact of humans to farm animals can increase the colonisation rate of humans with livestock associated methicillin-resistant *Staphylococcus aureus* (LA-MRSA) [18, 19]. More recently, it was postulated that an increased colonisation rate of humans with LA-MRSA is associated with higher infection rates [20]. One important tool used to investigate the dissemination of LA-MRSA was the typing of strains, especially the multilocus sequence typing (MLST). The application of MLST was also used to demonstrate for the first time the potential transmission of *S*. *gallolyticus* from laying hens to a farmer who suffered from infective endocarditis [21]. It seems that poultry flocks could be a reservoir of *S*. *gallolyticus*. However, studies on the dissemination of *S*. *gallolyticus* types in or between poultry flocks are missing although tools for the isolation, identification and typing of *S*. *gallolyticus* are available [22, 23]. Combining these tools may help to increase the knowledge concerning the occurrence of *S*. *gallolyticus* in farm animal herds and should provide the opportunity to compare animal and human isolates. Faecal samples from birds could be a useful source for detecting the bacterium [24].Therefore, the opportunity to take faecal samples from organic turkey flocks within the framework of a welfare project [25] was taken to obtain information about the occurrence and spread of *S*. *gallolyticus* in turkey flocks.

## Material and Methods

### Sampled Farms

Samples were taken from 18 organic turkey flocks from 10 different poultry farms located in the north, east and south of Germany. The term organic is defined in the Regulation (Ec) No 2160/2003 [26]. The farms housed Kelly Broad Breasted Bronze (Kelly BBB) turkeys, British United turkeys 6 (B.U.T 6) or Converter turkeys. Table 1 contains information about the sampled flocks. A flock is defined according to Regulation (EC) 2160/2003 of the European Parliament and Council [27].

**Table 1:** Characteristics of the sampled flocks.

Flock / farm	Located in [Federal state]	Sampled	Breeding line	Age of birds [weeks]	Flock size
**1 / 1**	Bavaria	September 2012	B.U.T. 6	6	1500
**2 / 2**	Mecklenburg-Western Pomerania	September 2012	Kelly BBB	18	1319
**3 / 2**	Mecklenburg-Western Pomerania	September 2012	Kelly BBB	18	1072
**4 / 3**	Saxony	September 2012	Kelly BBB	6	1800
**5 / 3**	Saxony	September 2012	Kelly BBB	18	200
**6 / 4**	Lower Saxony	October 2012	B.U.T. 6	4	1075
**7 / 5**	Lower Saxony	October 2012	Kelly BBB	15	800
**8 / 6**	Lower Saxony	November 2012	B.U.T. 6	17	1655
**9 / 6**	Lower Saxony	November 2012	B.U.T. 6	17	1600
**10 / 7**	Mecklenburg-Western Pomerania	November 2012	Kelly BBB	11	1750
**11 / 1**	Bavaria	November 2012	B.U.T. 6	16	1500
**12 / 1**	Bavaria	November 2012	B.U.T. 6	16	1500
**13 / 2**	Mecklenburg-Western Pomerania	November 2012	Kelly BBB	8	1314
**14 / 2**	Mecklenburg-Western Pomerania	November 2012	Kelly BBB	8	1100
**15 / 2**	Mecklenburg-Western Pomerania	November 2012	Kelly BBB	8	740
**16 / 8**	Saxony	November 2012	B.U.T. 6	16	1750
**17 / 9**	Lower Saxony	January 2013	Converter	9	933
**18 / 10**	Mecklenburg-Western Pomerania	January 2013	Kelly BBB	8	2340

### Sampling

Samples were taken between the beginning of September 2012 and the end of January 2013 (Table 1). At least five samples of individual fresh droppings were collected with gloved hands from randomly selected littered areas of each flock. New gloves were used for each fresh dropping. Faecal samples were collected in sterile plastic bags which were sealed and then transported under ambient temperature (in an air-conditioned car) to the laboratory for further processing within 24 h. The durations of transports ranged between two and seven hours. The samples were analysed either immediately after arrival at the laboratory or stored at 4°C for analysis within the next 24 h. All farm owners gave permission to conduct the study on their farms. Animals were not touched, replaced or used in any way to obtain the samples. As no animal experiments were carried out, an approval by a national ethics committee was unnecessary.

### Detection and quantification of *S*. *gallolyticus*

**Fig 1:**
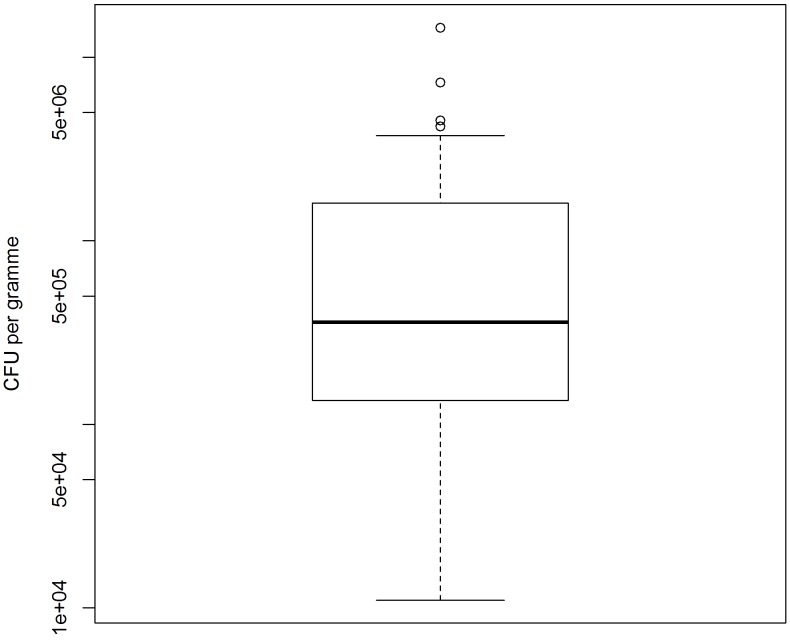
Logarithmic scaled Box-and-whisker plot with outliers of the *S*. *gallolyticus* subspecies *gallolyticus* concentrations from n = 39 faecal samples. Dots show outliers outside the 1.5-fold interquartile range.

For the qualitative detection of *S*. *gallolyticus* two grams of faeces of each sample (n = 26) from flocks 1/1, 2/2, 3/2, 4/3 and 5/3 (Table 1) were dissolved in four ml phosphate buffered saline (PBS with 0.01% v/v Tween 20) and incubated at room temperature for 30 minutes. After incubation samples were mixed for one minute at full speed on a vortex (MS 1 Multishaker, IKA® Works Inc., Wilmington, North Carolina, USA). Then a loop full of dissolved turkey faeces was streaked out directly onto Trypton Soya Agar (CM0131, Oxoid, LTD Basingstore, Hampshire, England) with final concentrations of 0.5% (w/v) tannin acid pure (A3619, Applichem, Germany) and 0.25 g/l of sodium azide pure (Merck, Germany). Chemicals were added to the medium to inhibit Gram-negative and Gram-positive bacteria including other streptococci than *S*. *gallolyticus* [28, 29]. Inoculated plates were incubated up to 48 h in a 5% CO_2_ atmosphere at 36°C. For the quantitative detection, all positive faecal samples that were not used for the qualitative analysis were investigated (n = 56). Therefore, 1 g of each faecal sample was diluted in 9 ml of PBS buffer and mixed for 1 minute on a vortex as described above. A serial dilution was prepared and 0.1 ml of each dilution step was distributed on a modified Trypton Soya Agar plate by means of the Drigalski-spatula technique. Only colonies looking identical to those of a growing control (Strain LMG 14622 from pigeon) were counted. Plates with a minimum of ten and a maximum of 400 presumed colonies were considered for quantification. The number of colonies per plate was multiplied by the dilution factor and related to the amount of faeces. Descriptive statistics on the concentrations (Fig 1) were performed by using the software R version 3.1.1 (2012-07-10, The R Foundation for Statistical Computing).

### Identification and typing of *S*. *gallolyticus*

From each sample used for the qualitative and quantification detection of *S*. *gallolyticus* one colony was streaked out on Columbia agar with 5% sheep blood (CM0331, Oxoid, LTD Basingstore, Hampshire, England). Plates were incubated aerobically at 36°C for 24 to 36 h. The isolate was analysed microscopically and biochemically by means of the catalase test (using 3% hydrogen peroxide), the oxidase test (Bactident, Merck, Germany) and the api® 20 STREP test (bioMérieux, Germany). Results of the api® 20 STREP test were analysed by the API 20 STREP V8.0 software available at https://apiweb.biomerieux.com/servlet/Identify?action=prepareNew&stripId=6 (accessed on 05/10/2015). In four cases the results of the api® 20 STREP test from the first isolated colony delivered an unacceptable identification. Therefore, a second colony from the same sample was used for identifying and typing *S*. *gallolyticus*. Isolates identified as *Streptococcus gallolyticus* subsp. *gallolyticus* were used to confirm the subspecies by MALDI-TOF mass spectroscopy and *sodA* sequence analyses as described by [23]. For the *sodA* sequence analysis, bacterial DNA was extracted using the QIAmp DNA Blood Kit (appendix, protocol D) (Qiagen, Hilden, Germany), according to the manufacturer’s instructions. Sequence analysis was performed as previously described [30].

**Fig 2:**
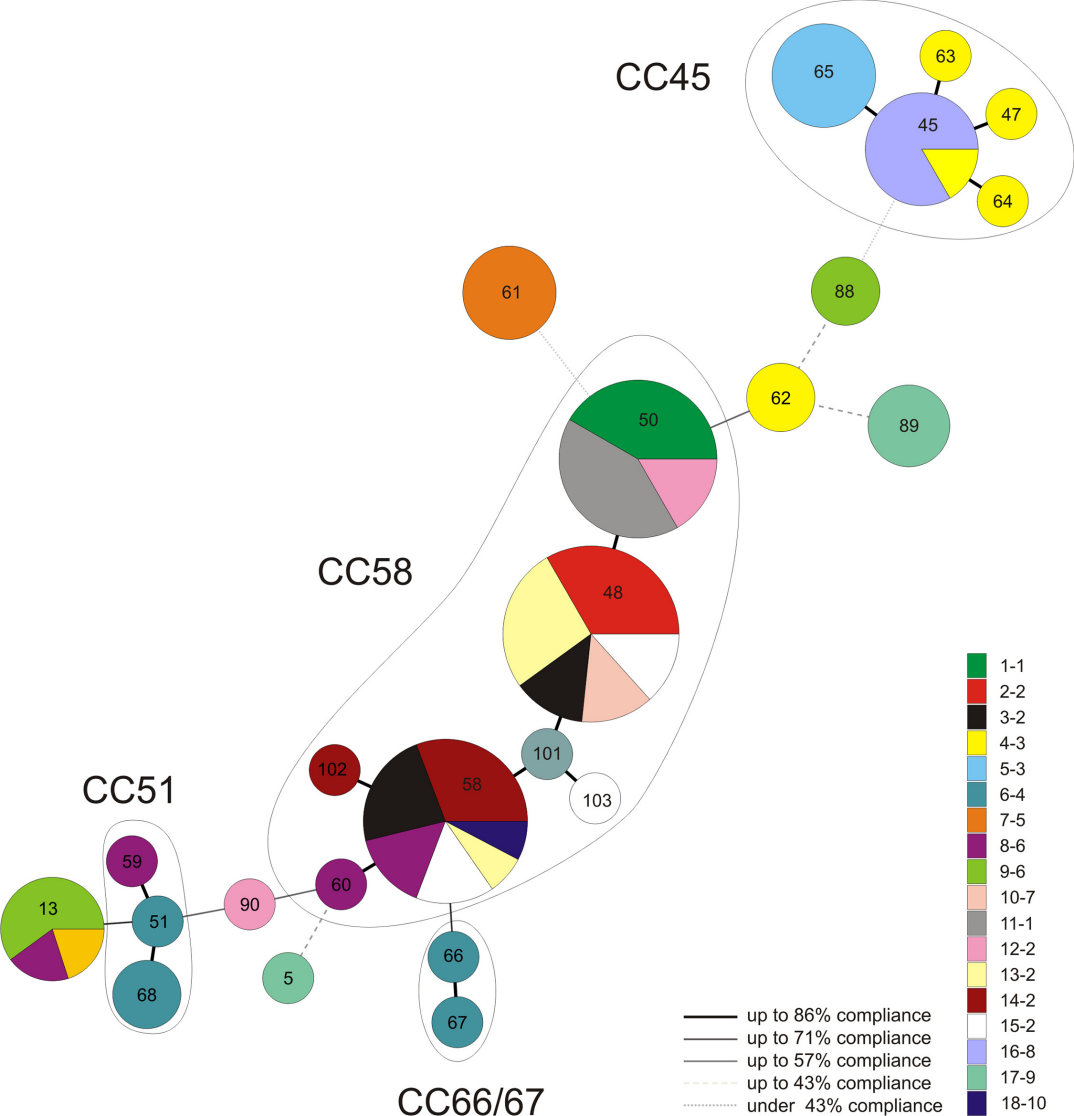
Clustering of 82 *S*. *gallolyticus* subspecies *gallolyticus* isolates from turkey flocks by use of MST. An MST was generated based on the UPGMA dendrogram (BioNumerics software 6.6 (Applied Maths, Sint-Martens-Latem, Belgium)). Each ST is shown as a circle, whose size is proportionate to the number of isolates. Colour codes represent the 18 flocks (and farm numbers) from which the STs were obtained. Lines linking the circles describe the compliance.

For typing, from each positive faecal sample one isolate identified as *S*. *gallolyticus* (n = 82) was analysed by multilocus sequence typing (MLST) as described by [31]. To examine sifnificant associations between STs and api® strip profiles Fisher's exact test was calculated with R. The relatedness of sequence types (STs) based on the UPGMA (Unweighted Pair Group Method with Arithmetic Mean) dendrogram (BioNumerics software 6.6 from Applied Maths, Sint-Martens-Latem, Belgium) is illustrated in a minimum spanning tree (MST, Fig 2). The clonal complexes (CCs) were determined by eBURST (eBURST version 3; most stringent definition (six out of seven alleles shared) www.mlst.net) [32]. Furthermore, to compare identified STs of turkeys with types from other animal species and humans the *Streptococcus gallolyticus* MLST website (http://pubmlst.org/sgallolyticus/) developed by Jolley and Maiden [33] was accessed on 04 August 2015.

### Estimation of the prevalence of *S*. *gallolyticus* in organic turkey flocks

The probable prevalence range of shedding turkeys was estimated by using the online software WinEpi, Working in Epidemiology, available at http://www.winepi.net/uk/index.htm (visited on 15/10/2014). For calculations the module “Disease measurements” and the submodule “Calculation of Prevalence” were used. The confidence level was set at 95%.

## Results

### Detection and quantification of *S*. *gallolyticus* in faeces

On all ten farms and in each of the 18 flocks *S*. *gallolyticus* was present at least in one of the faecal samples (Table 2). This also means that the bacterium was shed by three different breeding lines. The age of birds within the positive flocks varied between four and 18 weeks. Positive samples were taken during late summer, autumn or winter time. In most cases all faecal samples from one flock were tested positive for *S*. *gallolyticus*. In total, the bacterium was detected in 82 out of 91 investigated faecal droppings (detection frequency = 0.90).

**Table 2:** Sequence types of *S*. *gallolyticus* detected in faecal samples of 18 organic turkey flocks.

Flock / farm	No. of positive faecal samples	Detected sequence types
**1 / 1**	5	5 x ST50
**2 / 2**	5	5 x ST48
**3 / 2**	5	2 x ST48, 3 x ST58
**4 / 3**	6	ST45, ST47, 2 x ST62, ST63, ST64
**5 / 3**	5	5 x ST65
**6 / 4**	5	ST51, ST66, ST67, 2 x ST68
**7 / 5**	4	4 x ST61
**8 / 6**	5	ST13, 2 x ST58, ST59, ST60
**9 / 6**	5	3 x ST13, 2 x ST88
**10 / 7**	3	ST13, 2 x ST48
**11 / 1**	5	5 x ST50
**12 / 1**	3	2 x ST50, ST90
**13 / 2**	5	4 x ST48, ST58
**14 / 2**	5	4 x ST58, ST102
**15 / 2**	5	2 x ST48, 2 x ST58, ST103,
**16 / 8**	5	5 x ST45
**17 / 9**	5	ST5, 3 x ST89, ST101
**18 / 10**	1	ST58

Thirty-nine out of 56 faecal samples could be used for quantification. The other samples were not practicable due to growing and swarming of other bacteria or because the amount of presumed *S*. *gallolyticus* colonies per plate was too low for a reasonable quantification. Fig 1 summarises the results in a box-and-whisker plot with a 1.5 interquartile (IQR) range and outliers. Considering that the y-axis is logarithmic, the concentrations are non-normally distributed. The numbers of CFU of *S*. *gallolyticus* ranged between 1.1 × 10^4^ (lower quartile) and 1.6 × 10^6^ (upper quartile) per gramme. The median was 3.6 × 10^5^ CFU/g.

### Estimated prevalence range of shedding birds

Assuming that a positive fresh dropping originated from one bird the prevalence of shedding turkeys was estimated. The calculated true prevalence (95% confidence level) was 100% for all flocks with five positive out of five and six positive out of six samples, respectively. For four positive samples, the true prevalence was calculated between 45 and 100%, for three positive samples it was between 17 and 100%, and for one positive sample it ranged between 0 to 55%. The amounts of positive samples per flock are given in Table 2. This means that in 14 flocks (78% of the investigated flocks) the calculated prevalence of turkeys shedding *S*. *gallolyticus* was 100%.

### Typing and allelic profiles of *S*. *gallolyticus* isolates from faeces

At least one isolate per flock was typed by MLST. Table 2 shows the results of typing. The corresponding allelic profiles are shown in the supporting information (S1 Table). In total, 24 different STs were detected. In 11 out of 18 flocks, more than one ST was excreted. On farms 1 and 2, the same STs were isolated from the subsequently housed flock. Sequence types ST13, ST45, ST48 and ST58 could have been found on different farms and in flocks with different breeding lines. Furthermore, ST45 and ST58 were also found on farms located in different regions. Four clonal complexes determined by eBurst are shown in Fig 2. Most isolates (n = 44) belonged to the clonal complex CC58 found in 12 flocks deriving from six different farms. Fig 2 also illustrates that isolates from one flock could belong to the same CC (e.g. flock 15), to two CCs (e.g. flock 6) or to STs of high diversity partly from the determined CCs (e.g. flock 17). A comparison of all isolated STs from this study with uploaded types on the MLST website revealed that four STs were already found in other species. Sequence type ST45 was isolated from a pigeon, ST5 and ST48 were isolated from humans and ST13 was isolated from chickens and a human patient with endocarditis.

### api® strip profiles

Sixty-eight presumptive *S*. *gallolyticus* isolates were analysed by using the api® 20 STREP test. In 82 cases *Streptococcus gallolyticus* subsp. *gallolyticus* was clearly identified (id% 95.8–99.9). Three times the first taxon was *Aerococcus viridans* and in one case the result was *Globicatella sanguinis*. All profiles of *S*. *gallolyticus* isolates are included in the supporting information (S1 Table). In total, 10 different biochemical profiles were found among all isolates. Sixty-four isolates (78% of all typed isolates) showed the profile 5 0 4 0 5 5 3. This profile was detected in 21 different STs (S1 Table) and in 89% of isolates from CC58. Two to three different biochemical profiles from the same ST (ST45, ST50, ST58, ST61, ST62 ST65, ST88 and ST89) were observed in eight cases. For instance, ST58 (13 isolates, three profiles) seems to be more variable regarding the biochemical reactions as ST48 (15 isolates, one profile). No significance of the difference between the two proportions can be assessed by using Fisher's exact test (p = 0.206).

## Discussion

*Streptococcus gallolyticus* can be an infectious agent in both animals and humans. However, there is only limited information about the occurrence of this bacterium in farm animals and the adaption of this species to its host. Furthermore, there is an on-going discussion concerning the possibility of transmission between different animal species and between animals and humans [34]. To obtain more information about the spread of this bacterium easy and efficient isolation techniques would be helpful. In this study, a modified solid medium was used which allows the detection of *S*. *gallolyticus* by streaking out a faecal sample dilution from birds without any preselecting steps. As Osawa and Mitsuoka [35] hypothesised, the ability of *Streptococcus bovis* biotype I (today classified as *S*. *gallolyticus*) to degrade tannin-protein complexes could be useful to isolate the bacterium from animal faeces. Although these authors solved the problem of inhibiting the massive growth of Enterobacteria by using antibiotics, the media they produced failed to enumerate *Streptococcus bovis*. Therefore, a different medium for the plates was applied in the present study, and sodium azide was added instead of antibiotics to inhibit the Gram-negative microbiota. The growth of bacteria other than *S*. *gallolyticus* was observed in some cases, disturbing the counting on the modified media. Nevertheless, in 39 out of 56 cases the inoculated media could be used for counting *S*. *gallolyticus* directly from dissolved faecal samples. Considering the variety of different cultivable species in faeces from turkeys [36] the used medium showed an acceptable selectivity. This selectivity was confirmed by identifying 82 out of 86 (95%) presumed *S*. *gallolyticus* isolates from different samples by using biochemical tests, PCR and MALDI-TOF. This also indicates that the mistake of counting the presumed colonies is probably low. For instance, one false positive out of twenty presumed isolates reveals statistically a *S*. *gallolyticus* prevalence of 85% by a given probability of 95% [37].

*Streptococcus gallolyticus* seems to be widespread among turkeys in organic husbandries. All examined flocks from ten farms in different regions of Germany keeping different turkey breeding lines were positive. None of the flocks showed symptoms of an acute or chronic streptococcosis. If single cases of infections or secondary infections occurred remains unknown because no pathological examinations of dead birds were carried out in this study. Concerning the spread of *S*. *gallolyticus* or the formerly named *Streptococcus bovis* biotype I in turkey production, only limited data are available from literature. One case report described *S*. *bovis* infections in two breeder facilities and one flock of fattening turkeys in the United States of America [10]. The agent was isolated from the spleen, liver and bone marrow of infected birds. However, although the authors used the api® 20 STREP test for identification, no information was given about the biotype and it remains unclear whether or not *S*. *gallolyticus* was isolated.

Besides the obvious spread of *S*. *gallolyticus* on organic turkey farms, this study also indicated a high prevalence within colonised flocks. The investigated fresh faecal droppings originated from different locations of one animal house. Therefore, it is unlikely that different droppings were shed by the same bird. The total detection frequency of 0.90 and the statistically estimated prevalence of birds shedding *S*. *gallolyticus* support the conclusion of high prevalence of *S*. *gallolyticus* among turkeys in organic husbandries. To the best of our knowledge this is the first information about the prevalence of *S*. *gallolyticus* in poultry flocks. As turkey lines such as B.U.T. 6 and Converter are also kept in conventional systems it seems possible that *S*. *gallolyticus* is also present in conventional husbandries. However, the kind of husbandry system and different management practice may influence the occurrence of bacteria species within poultry flocks [38].

The dynamics of bacteria occurrence in laying hen flocks for instance was described by repeated measurements during a laying period [39]. In the present study, no repeated samplings were conducted within the same flocks. However, already in younger flocks (e.g. flocks no. 1, 4 and 6) a high prevalence of *S*. *gallolyticus* was estimated, and the species was also present in different flocks (e.g. 2, 3, and 6) at the end of the fattening period. Positive faecal shedding from birds of different ages suggests that *S*. *gallolyticus* belongs to the persistent microbiota in the turkey gut. The median of CFU detected in turkey faeces, for example, is comparable to averages of *Enterococcus* spp. which are also group D streptococci and probably belong to the common intestinal microbiota of turkeys [40, 41]. Routes of entry may be, for example, poultry feed, explaining an early colonisation [36]. The age of faecal samples and transport conditions may impact the bacterial content of faeces. However, fresh droppings were sampled and it is believed that transport conditions had no significant impact on the measured CFU. For instance, Cools et al. [42] showed significant influences of different temperatures (between 5°C and 25°C) and different moisture contents on the number of faecal Streptococci in fresh manure only days later.

One intention of this study was to contribute to the discussion about the possible transmission between different animal species and between animals and humans. MLST could be a powerful epidemiological tool to investigate the transmissions of infectious agents or zoonotic pathogens [43, 44]. Dumke et al. [31] developed a MLST to type *S*. *gallolyticus* which was used in this study. In total, 24 different sequence types were detected. The MST shows that most of the flocks harboured either one ST or STs belonging to the same CC. More than 50% of all isolates were part of CC58. Sequence types of this complex were obtained from flocks of different regions and from varying breeding lines. This could mean that STs of CC58 are dominant in turkey production. Interestingly, 89% of the isolates from CC58 showed the same biochemical profile. Although, no significant association between phenotypes and STs were found in this study, the results do not contradict a genetic homogeneity within CC58. Anyway, further studies are necessary to verify this assumption.

All in all, the MST reflects a heterogenic population of *S*. *gallolyticus* in the investigated turkey flocks. In some cases, e.g. flocks 9, 10 and17, STs with very low compliance were found simultaneously in the same flock. Results of the api® 20 STREP test indicated that in some cases the same ST differed in its phenotype. This may be a hint of genetic variations between the same STs. On the other hand, ST13, ST45, ST48 and ST58 were found on different farms and isolates of the same ST did not differ in its biochemical reactions. Interestingly, ST5, ST48, ST45 and ST13 found in faeces in the present study had already been isolated from other species. Sequence types five and 48 had been isolated from humans, ST45 from a pigeon and ST13 from chickens and a human patient. Dumke et al. [21] reported that this patient had contact to hens carrying the same sequence type. Of course, these results do not implicitly prove the transmission between turkeys and other species but nevertheless provide first hints at the possibility thereof. For instance, the transmission of Gram-positive, facultative anaerobic pathogenic bacteria from the poultry gut to poultry farmers has already been suggested by Van den Bogaard et al. [45]. Furthermore, Ellmerich et al. [46] showed that a human isolate of *Streptococcus bovis* biotype I could act as a promoter of early preneoplastic lesions in the colon of rats. Lin et al. [3] sequenced a human strain with a genome that is more adapted to ruminal environments. Thus, an interspecies transmission cannot be ruled out. To analyse this in detail, further studies on the transmission between different species and the potential of *S*. *gallolyticus* animal isolates to cause infection in humans are needed. In the present study the main objective was to acquire information about the spread of *S*. *gallolyticus* in turkey flocks, farmers being excluded from the study. However, the applied method to isolate *S*. *gallolyticus* from turkey faeces is also useful to isolate the bacterium from human stool samples (Isolates HDZ 1166 and HDZ 1167 published in the MLST database (http://pubmlst.org/sgallolyticus/)). This may enable the investigation of the association between isolates from poultry and humans in future studies.

## Conclusion

The used methods in this study can help to obtain more information about the spread of *S*. *gallolyticus* in animals and potential transmission risks. The high detection rate and the number of *S*. *gallolyticus* in turkey faeces indicate that this bacterium probably belongs to the common microbiota of the gastrointestinal tract of turkeys from organic flocks. Typing results suggest a predominant CC. However, four STs isolated in the present study were detected in other species of earlier published studies. Hence, an interspecies transmission cannot be ruled out and further studies should clarify whether farm animals can be a source of human infections.

## Supporting Information

S1 Table Sequence types, allelic profiles and api® strip profiles from *Streptococci gallolyticus* subsp. *gallolyticus* isolates. (XLSX)
